# Optimization of Milk-Based Medium for Efficient Cultivation of *Bifidobacterium pseudocatenulatum* G4 Using Face-Centered Central Composite-Response Surface Methodology

**DOI:** 10.1155/2014/787989

**Published:** 2014-01-12

**Authors:** Khalilah Abdul Khalil, Shuhaimi Mustafa, Rosfarizan Mohammad, Arbakariya Bin Ariff, Yamin Shaari, Yazid Abdul Manap, Siti Aqlima Ahmad, Farrah Aini Dahalan

**Affiliations:** ^1^Department of Biomolecular Sciences, Faculty of Applied Sciences, Universiti Teknologi MARA, Sec. 2, 40150 Shah Alam, Selangor, Malaysia; ^2^Halal Products Research Institute, Universiti Putra Malaysia, Putra Infoport, 43400 Serdang, Selangor, 40150 Shah Alam, Selangor, Malaysia; ^3^Faculty of Biotechnology and Biomolecular Sciences, Universiti Putra Malaysia, 43400 13 Serdang, Selangor, Malaysia; ^4^Institute of Tropical Forestry and Forest Products, Universiti Putra Malaysia, 43400 15 Serdang, Selangor, Malaysia; ^5^Faculty of Food Science and Technology, Universiti Putra Malaysia, 43400 Serdang, Selangor, Malaysia; ^6^The School of Environmental Engineering, Universiti Malaysia Perlis, Kompleks Pengajian Jejawi 3, 02600 Arau, Perlis, Malaysia

## Abstract

This study was undertaken to optimize skim milk and yeast extract concentration as a cultivation medium for optimal *Bifidobacteria pseudocatenulatum* G4 (G4) biomass and **β**-galactosidase production as well as lactose and free amino nitrogen (FAN) balance after cultivation period. Optimization process in this study involved four steps: screening for significant factors using 2^3^ full factorial design, steepest ascent, optimization using FCCD-RSM, and verification. From screening steps, skim milk and yeast extract showed significant influence on the biomass production and, based on the steepest ascent step, middle points of skim milk (6% wt/vol) and yeast extract (1.89% wt/vol) were obtained. A polynomial regression model in FCCD-RSM revealed that both factors were found significant and the strongest influence was given by skim milk concentration. Optimum concentrations of skim milk and yeast extract for maximum biomass G4 and **β**-galactosidase production meanwhile low in lactose and FAN balance after cultivation period were 5.89% (wt/vol) and 2.31% (wt/vol), respectively. The validation experiments showed that the predicted and experimental values are not significantly different, indicating that the FCCD-RSM model developed is sufficient to describe the cultivation process of G4 using skim-milk-based medium with the addition of yeast extract.

## 1. Introduction

Probiotic can be defined as “living microorganisms which when administered in adequate amounts confer a health benefit on the host” [1]. The beneficial effects of probiotic include regulation in immune system, anticarcinogenic, preventing the infection by exogenous microorganisms, vitamin synthesis, and enhancing digestion and absorption of nutrients [2].

Bifidobacteria, the major species of intestinal flora in healthy human, have been widely used in probiotics formulation [3]. Bifidobacteria are anaerobic rods, Gram-positive, and nonspore forming bacteria [4]. *Bifidobacterium pseudocatenulatum* G4, isolated from breast-fed infants feces, has been identified and characterized as a potential probiotic with many beneficial health effects [5–7]. The antimicrobial properties, adherence ability, antagonistic activity, and ability to survive in gut environment for *Bifidobacterium pseudocatenulatum* G4 have also been reported [8].

Proliferation of bifidobacteria requires appropriate medium which supplies nurturing substances for growth. The use of commercial media to support bacterial growth is limited by its cost [9] and off-flavor generated in food products [10]. Milk is a highly nutritious growth medium for many microorganisms as it is rich in carbohydrates, fat, casein, protein, vitamins, and minerals [11]. However, some microorganisms are unable to grow well in this medium when specific enzymes required for lactose metabolization process are lacking [12]. *β*-Galactosidase is an enzyme required to break lactose to galactose and glucose. Most of the bifidobacteria preferred these carbon sources to promote their growth. The production of *β*-galactosidase can be enhanced using yeast extract as a nitrogen source [13]. Various concentrations of yeast extract (ranging from 0.3% to 1.0% wt/vol) were added into milk (ranging from 10% to 12% wt/vol) to enhance growth of probiotics [14, 15]. Various concentrations of glucose (ranging from 0.5% to 2.0% wt/vol) have also been used as a carbon source in the cultivation of probiotic like bifidobacteria [16].

In optimization of milk-based-medium for bifidobacteria growth, it is important to consider *β*-galactosidase production by the cells in order to facilitate lactose breakdown into more readily available sugar. In addition, the concomitant use of nitrogen sources is also important to avoid nutrients wastage. Most of the previous studies on the medium optimization for bifidobacteria focused mainly on the maximum biomass production [8, 17]. To the best of our knowledge, justification involving other responses of growth activities of bifidobacteria in milk medium in order to ensure that the medium has been used competently is not available in the literature.

The optimization of medium concentration or components by conventional approach involves large numbers of experiments which are time consuming and expensive and lack in representing the effects of individual factors. Thus, a statistical approach known as response surface methodology (RSM) has been applied as a tool for process improvement. The main advantage of RSM is the reduced number of experiments or trials required to evaluate multiple parameters and their interactions [18, 19]. The most commonly design approached is the central composite design. The design allows estimation of all the regression parameters required to fit a second-order model of given responses. Rotability character is the most preferred in any central composite design. This is because this characteristic provides constant variance of the estimated response corresponding to all new observation points that are at the same distance from the center point of the design (in terms of the coded variable).

Therefore, the main objective of this study was to optimize milk-based medium for the improvement of *B. pseudocatenulatum* G4 cultivation. In this optimization process, there were four responses; namely, (i) cells production, (ii) accumulation of lactose degrading enzyme (*β*-galactosidase), and (iii) residual concentration of lactose and free amino nitrogen remaining in the culture at the end of cultivation, were considered.

## 2. Materials and Methods

### 2.1. Bacterium and Inoculum Preparation

Probiotic bacterium,* B. pseudocatenulatum* G4 (G4), was used throughout this study and this bacterium was obtained from the Probiotic Laboratory, Faculty of Food Science and Technology, Universiti Putra Malaysia. G4 was previously isolated from breast-fed infant feces [5, 6] and was stored at −20°C in a mixture of glycerol and Trypticase Phytone Yeast (TPY) extract broth (Scharlau-Chemie, Barcelona, Spain), at a ratio of 80 : 20. TPY medium (Scharlau-Chemie, Barcelona, Spain) was used to maintain and to propagate the bacterium [4]. A single colony of the bacterium was transferred from TPY agar to TPY broth and incubated anaerobically at 37°C for 24 h. After two successive transfers in TPY broth at 37°C for 24 h under anaerobic condition using Anaerocult A gas packs (Merck, Darmstadt, Germany), the activated culture was then properly diluted and served as the standard inoculum for all cultivations.

### 2.2. Medium Preparation

Cultivation was carried out in 250 mL SCHOTT DURAN bottle (Schott Duran, Mainz, Germany). The medium components consist of skim milk (NZMP medium heat skim milk powder, Auckland, New Zealand), yeast extract (Bio Springer, Maisons-Alfort Cedex, France), and glucose (Merck, Darmstadt, Germany). All components were prepared in separate bottle and sterilized at 121°C for 15 min. Once cooled at room temperature, the components were mixed prior to inoculation with 10^4^ cfu/mL of G4. The bottle was then incubated at 37°C for 21 h in an anaerobic condition. The culture pH was not controlled in all experiments. During the cultivation, 10 mL of samples was taken at 3 h intervals for analysis.

### 2.3. Lactose Determination

The amount of lactose in the samples was determined using HPLC method as described by Hou et al. with some modifications [20]. One mL of sample was centrifuged at 13000 rpm for 10 min. The clear fraction was filtered through a 0.2 *μ*m nylon membrane filter and injected onto HPLC system (Alliance 2690/5: Waters Corporation, Milford, CA), equipped with a 4.6 mm × 150 mm Agilent Zorbax Carbohydrate Analysis column (Agilent Technologies Inc., USA). The mobile phase used is 75% (v/v) acetonitrile (Fisher, HPLC grade). The flow rate was set at 1.4 mL min^−1^ and the analysis was carried out at 30°C using a refractive index detector (RI-1371, Waters Corporation, Milford, CA).

### 2.4. Free Amino Nitrogen Analyses

Free amino nitrogen (FAN) presence in the sample was determined using ninhydrin analysis described by Hwang and Ederer with some modifications [21]. A sample (1 mL) was centrifuged at 13000 rpm for 10 min. The clear fraction was collected and diluted into 50 mL distilled water and 2 mL of the diluted sample was then transferred into test tubes. Ninhydrin solution (0.35 g ninhydrin into 100 mL of a 1 : 1 mixture of acetone and butanol) was then added and the tubes were covered with a piece of parafilm to avoid the loss of solvent due to evaporation. The tubes were then heated in a boiling water bath (80°C–100°C) for 15 min before being transferred to a cold water bath. The dilution reagent (5 mL) was then added and mixed and the absorbance was read at 570 nm using spectrophotometer (UV-Vis 1601 spectrophotometer, Shimadzu, Japan). Distilled water was used as a blank and glycine (Merck, Darmstadt, Germany) was used as a standard.

### 2.5. *β*-Galactosidase Analyses

*β*-Galactosidase was assayed on nonpermeabilized and permeabilized cells. The culture was centrifuged at 11000 rpm for 10 min at 4°C. Subsequently, the resulting supernatant was discarded by aspiration. The cell pellet was washed twice with Z buffer (0.1 M phosphate—pH 7; 10 mM MgSO_4_·7H_2_O; 1 mM CaCl_2_) and concentrated by 10-fold. Permeabilization was accomplished by the addition of 0.5 mL of Triton X-100 (5% vol/vol in Z buffer) into 0.5 mL cell suspension with mixing using vortex mixer. The mixture was incubated at 37°C for 10 min and then centrifuged at 14000 rpm for 15 min and the resulting supernatant served as the enzyme source. The activity of *β*-galactosidase was then assayed essentially according to the method described by Nagy et al. (2001) [22]. The reaction mixture was composed of 0.5 mL of enzyme sample and 0.5 mL of 15 mM o-nitrophenyl *β*-d-galactopyranoside (Sigma Chemical Co., St. Louis, MO) in 0.03 M sodium phosphate buffer (pH 6.8). Once a faint yellow tint appeared, the reaction was terminated by adding 0.1 M sodium carbonate. The absorbance was measured using a spectrophotometer at 420 nm wavelength (UV-Vis 1601 spectrophotometer, Shimadzu, Japan). One unit of *β*-galactosidase activity was defined as the amount of enzyme catalyzing the formation of 1 *μ*mol of o-nitrophenyl per min under the assay condition.

### 2.6. Microbiological Analyses

For viable cells enumeration, samples were serially diluted using 0.1% (wt/vol) sterile peptone water (Merck, Darmstadt, Germany) and plated in duplicate onto TPY agar. The plates were incubated in anaerobic jars containing Anaerocult A (Merck, Darmstadt, Germany) at 37°C for 48 h. All plates with 30 to 300 colonies were counted and the viability was expressed as log_10_ cfu/mL.

### 2.7. Experimental Design and Statistical Analyses

Design-Expert software (Stat-Ease Inc., Minneapolis, MN, USA, version 6.0.6) was used to design the experiment in this study. First, initial screening experiments were performed to evaluate the significant effects of skim milk, yeast extract, and glucose on biomass production using a 2^3^ full factorial design. Each factor was coded at three levels between −1 and +1, with 5 times replication of center point (0), where the variables of factors were changed in the ranges. The factorial design resulting in 26 experimental runs (including duplicates and 5 middle points run). In the second step, a first-order empirical equation was applied to exclude insignificant factors and to generate the steepest ascent path to facilitate the maximum increase in response. The steepest ascent design was based on results of the equation from the screening step of which only factors with significant effects on response were used and fixed at their zero coded value. The insignificant factors were eliminated from the design. The path begins at the centre point of design and stretches out linearly in order to determine the suitable ranges within significant factors which tend to approach the optimal condition. The third step involved further optimization of significant factors using face-centered central composite design (FCCD). FCCD is an effective design that is used for sequential experimentation and provides reasonable amount of information for testing the goodness of fit and does not require large number of design points thereby reducing the overall cost associated with the experiment [23]. In this design, four responses, namely, biomass (*y*_1_, log_10_ cfu/mL) and *β*-galactosidase production, (*y*_2_, U/mL) as well as residual lactose (*y*_3_, g/L) and FAN (*y*_4_, mg/L) remaining in the culture were determined. There were three coded factor levels, −1, 0, and +1 (where −1 corresponded to the low level of each factor, 0 to the middle level, and +1 to the high level). The coded values were determined using

(1) coded  value  =actual  level−(maximum  level−minimum  level)(1/2)(maximum  value−minimum).

The center point was repeated five times in order to evaluate the curvature and the experiment replication facilitated the pure error estimation, so that the significant lack of fit of the models could be predicted. All the 26 experiments were carried out in duplicate for 21 h.

## 3. Results and Discussion

### 3.1. Initial Screening of Significant Medium Components and the Steepest Ascent

The effect of skim milk (*x*_1_), yeast extract (*x*_2_), and glucose (*x*_3_) on biomass production (*y*_1_) during the cultivation of G4 using 2^3^ full factorial design is shown in Table 1. The maximum biomass (ranging from 4.495 to 7.638 log_10_ cfu/mL) was obtained at 21 h of cultivation. The analysis of variance (ANOVA) of the first-order model is shown in Table 2 while the regression analysis of the model is shown in Table 3. The model was significant and only 1.12% of the total variation was observed due to noise. The model was linear with insignificant (*P* > 0.05) curvature. The regression analysis of the variables showed that skim milk (*P* = 0.007) was the only significant factor while yeast extract and glucose were insignificant with *P* values higher than 0.05 (Table 3). Since yeast extract (*x*_2_) was the sole nitrogen, it must be considered in the medium optimization. Improvement of growth of bifidobacteria could be achieved using yeast extract at concentration higher than 1.0% without the addition of glucose [17]. As a result, final-order equation (coded term) was generated based on the first-order model to determine the biomass production response (*y*_1_) to the medium composition containing skim milk (*x*_1_) and yeast extract (*x*_2_) factors, which give

(2) $$\begin{array}{c} y_{1}=6.2+1.09x_{1}+0.3x_{2}. \end{array}$$

For every unit increase in *x*_1_, an increase of 1.09 units in *y*_1_ is predicted (2). On the other hand, for every unit increase in *x*_2_, 0.30 units of increase in *y*_1_is expected. Between these two factors, skim milk (*x*_1_) gave more effect than yeast extract (*x*_2_) on biomass production (*y*_1_). This equation was further used as the fundamental scale in the subsequent step which was the steepest ascent. The path of the steepest ascent was determined based on the increase in 0.10% (wt/vol) of *x*_1_ and the movement was generated along the path until no improvement occurred. Five design units were developed based on 0.10/0.10 = 1. Hence, the movement of *x*_2_ was 0.28 design units (0.3/1.09 = 0.28). As for natural factor, *x*_1_ movement was based on coded factor × lower point of *x*_1_ (1 × 2 = 2). Thus, movement of *x*_2_ natural factor involved 0.28 × 0.5 = 0.14. The path coordination of the steepest ascent was generated and is shown in Table 4. The highest biomass production can be observed in the third step of the steepest ascent coordinates with the value of 7.68 ± 0.17 log_10_ cfu/mL from the combination of skim milk (6% (wt/vol)) and yeast extract (1.89% (wt/vol)). After the third step of coordinates, declining in biomass production was observed. Consequently, this combination was selected as the middle point for further optimization.

### 3.2. Optimization of Medium Component

The experimental responses for the optimization of skim milk and yeast extract are shown in Table 5. Skim milk concentration (*x*_1_) was in the range of 4% (wt/vol) to 8% (wt/vol) with 6% (wt/vol) as a center point whereas, for yeast extract (*x*_2_), the range of 1% (wt/vol) to 2.8% (wt/vol) with 1.9% (wt/vol) as a center point was fixed further optimization. Center points with a coded (0,0) were repeated five times. The importance of medium components for cultivation process can be considered by their effect on biomass production (*y*_1_) and *β*-galactosidase production (*y*_2_) as well as the residual lactose (*y*_3_) and free amino nitrogen (*y*_4_) remaining in the culture. The responses (*y*_1_, *y*_2_, *y*_3_, and *y*_4_) were fitted with quadratic polynomial model and subsequently produced the response surface as expressed in (3). Consider

(3) $$\begin{array}{c} y=\beta_{0}+\beta_{1}x_{1}+\beta_{2}x_{2}+\beta_{11}x_{1}^{2}+\beta_{11}x_{2}^{2}+\varepsilon_{i}, \end{array}$$

where *x*_1_ and *x*_2_ represent coded independent factors of skim milk and yeast extract, respectively. Meanwhile, *β*_0_, *β*_1_, *β*_2_, *β*_11_, and *β*_22_ are coefficients and *ε*_*i*_ is the random error.

#### 3.2.1. Optimization of Biomass Production (*y*_1_)

By applying regression analysis on the experimental data, biomass production can be described by the second-order equation

(4) y1=7.14+1.01x1−6.67×10−3x2−0.93x12−0.023x22−0.055x1x2.

Skim milk (*x*_1_, *x*_1_^2^) was shown to be highly significant (*P* < 0.001) whereas yeast extract (*x*_2_, *x*_2_^2^) presented less influence towards biomass production. As a result, the quadratic model was further reduced with insignificant model term excluded. However, the addition of yeast extract (*x*_2_), as nitrogen source, in the milk medium must be considered to enhance growth of bifidobacteria [13]. Therefore, yeast extract is included in (4) to (5). Consider

(5) $$\begin{array}{c} y_{1}=7.14+1.01x_{1}-6.67\times 10^{-3}x_{2}-0.93x_{1}^{2}-0.055x_{1}x_{2}. \end{array}$$

Table 6 shows ANOVA result of quadratic model. Biomass production is significant as indicated by *P* value (*P* < 0.001). The *R* ^2^ implies the sample variation of 99.93% for biomass production (*y*_1_) and this indicates that only about 0.07% of total variation is not explained by the model, indicating good agreement between the experimental and predicted values for biomass production. The lack of fit measures the failure of the model to represent data in the experimental domain at points, which are not included in the regression [24]. The value of lack of fit for regression is insignificant (*P* > 0.05), suggesting that the model fitted well to the data in the experimental region. Moreover, second-order terms were found sufficient and higher order terms were not necessary.

**Figure 1:**
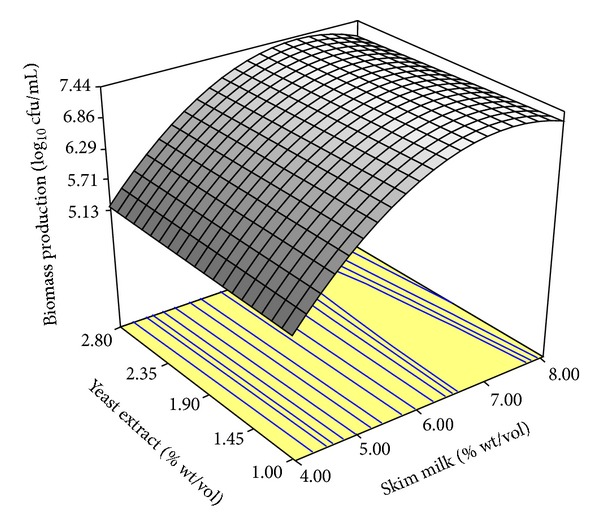
Response surface methodology for biomass production (log_10_ cfu/mL) from the adjusted quadratic mathematical model.

The response surface model shown in Figure 1 indicated that skim milk concentration (% wt/vol) was the most important factor in the medium. Lactose in milk was able to supply readily utilizable carbon for growth of G4. Increased milk concentration, with small addition of yeast extract as growth enhancer, significantly increased biomass production. Lactose in milk induced *β*-galactosidase biosynthesis in *Bifidobacterium* strain, which break down lactose into glucose and galactose more efficiently during the cultivation process [13]. The response surface obtained is a stationary edge system, whereby the maximum value is a plane platform rather than point form. Thus, there is flexibility in choosing the appropriate optimum points [25].

Based on the optimum point acquired from response surface methodology, the combination of 7.17% (wt/vol) skim milk, *x*_1_, and 1.02% (wt/vol) yeast extract, *x*_2_, was predicted to produce 7.44 log_10_ cfu/mL biomass. This prediction was verified by a validation experiment. A maximum biomass of 7.35 log_10_ cfu/mL was obtained from replication experiments. Even though the experimental value was lower than the predicted value, no statistical difference (*P* > 0.05) was observed. In order to observe other growth activities of G4 in optimized medium, analysis of *β*-galactosidase production as well as determination of lactose and free amino nitrogen residual in the culture at 21 h of cultivation was performed. As shown in Table 7, the optimized medium (7.17% (wt/vol) skim milk and 1.02% (wt/vol) yeast extract) was able to support G4 growth up to 7 log_10_ cfu/mL with substantially high *β*-galactosidase production at 21 h of cultivation. However, high amount of lactose and free amino nitrogen residual was still observed at the end of cultivation. To avoid wastage of nutrients remaining in the medium, further optimization step was carried out by considering residual nutrients remaining in the culture at the end of cultivation. This step was conducted and presented in the following section.

#### 3.2.2. *β*-Galactosidase Production (*y*_2_), Lactose (*y*_3_), and Free Amino Nitrogen (*y*_4_) Presence as Responses

Further optimization of G4 medium cultivation was carried out by including *β*-galactosidase production, lactose, and free amino nitrogen balance at 21 h of cultivation as responses. The responses (*y*_2_, *y*_3_, and *y*_4_) were fitted with the second-order polynomial equations

(6) y2=8.87+2.19x1+0.57x2−3.97x12−0.88x22−0.94x1x2,y3=9.64+5.02x1−0.15x2,y4=235.68+45.95x1+121.79x2+12.64x12+7.01x22+4.05x1x2.

The statistical significance of the model was evaluated by the *P* value of the analysis of variance (ANOVA). ANOVA statistics for the three responses, *y*_2_ (*β*-galactosidase), *y*_3_ (lactose residual), and *y*_4_ (free amino nitrogen residual), at 21 h of the cultivation period are shown in Table 8. Quadratic models for *y*_2_ and *y*_4_ as well as linear model for *y*_3_ are highly significant, as shown by low probability value (*P* < 0.001). The models fitted well to the experimental design as lack of fit for all three responses is insignificant (*P* > 0.05). Moreover, the coefficient of determinations (*R* ^2^) is close to 1 (*y*_2_: 0.97, *y*_3_: 0.99, and *y*_4_: 0.98), indicating high degree of correlation between observed and predicted values.

**Figure 2:**
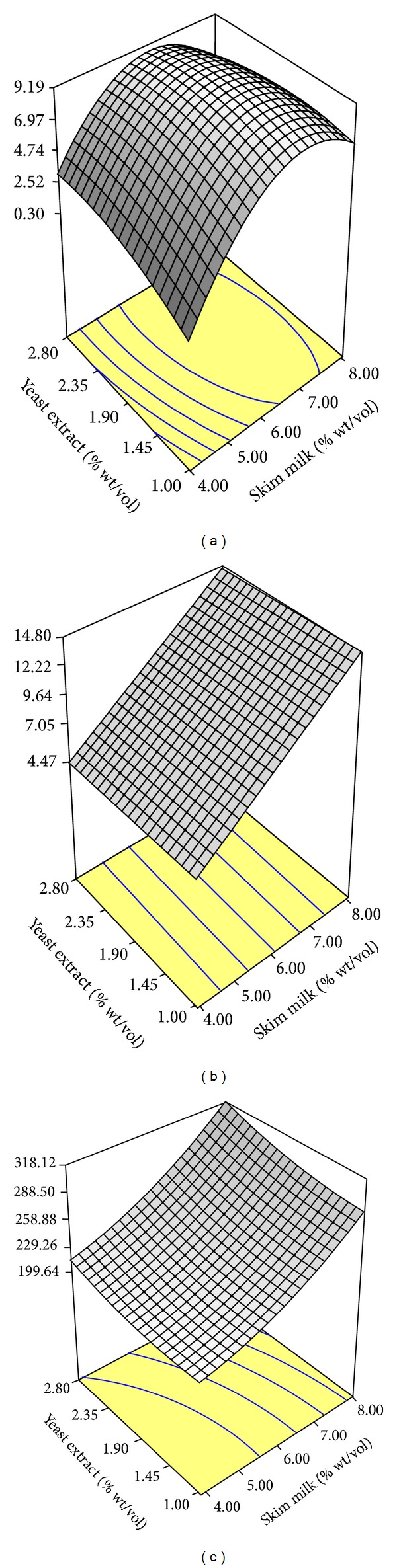
Response surface plot of the effect of skim milk and yeast extract concentration (% wt/vol) for the responses of (a) *β*-galactosidase production (U/mL), (b) lactose residual (g/L), and (c) free amino nitrogen residual (mg/L) at 21 h of cultivation period.

The response surface plot of *β*-galactosidase production (*y*_2_) is shown in Figure 2(a). High production of *β*-galactosidase was observed at the middle range of skim milk (6.00 to 7.00% (wt/vol)). On the other hand, reduced production of *β*-galactosidase was detected at high and low point of skim milk concentration. The use of high skim milk concentration (8.00% (wt/vol)) might contribute to the presence of high lactose concentration and this could suppress *β*-galactosidase production. Medium containing high lactose concentration (>5% [wt/vo]), may attributed to increase in the concentration of internally glucose. It has been reported that the increased of internal glucose would suppressed the biosynthesis of *β*–galactosidase by tested organisms [26]. The presence of low lactose concentration in the medium using low skim milk concentration (4.00% (wt/vol)) may not induce *β*-galactosidase production.

A linear response surface plot of lactose residual (*y*_3_) is shown in Figure 2(b). At high skim milk concentration (8.00% (wt/vol)) residual lactose concentration at 21 h of cultivation reached a maximum value with minor changes in yeast extract (1% to 2.8% (wt/vol)). In contrast, very low residual glucose concentration was detected at low skim milk concentration (4.00% (wt/vol)). This response indicated that skim milk concentration (4% to 8% (wt/vol)) was closely associated with residual lactose concentration. On the other hand, yeast extract ranging from 1% to 2.8% (wt/vol)) had less significant effect on the response. However, the presence of yeast extract was required to support *β*-galactosidase production (Figure 2(a)). Minimum residual lactose concentration at 21 h of cultivation was considered in this optimization method to avoid wastage of carbon source. From this point of view, low skim milk concentration is preferred to be used as cultivation medium for G4.

Figure 2(c) shows response surface plot representing free amino nitrogen residual concentration in the culture at 21 h of cultivation. The presence of nitrogen in medium was attributed to yeast extract and skim milk as well as dead cells and was believed to sustain bacterial growth. Similar to lactose, minimum residual concentration of nitrogen remaining in the culture after 21 h was considered in the optimization to avoid wastage of nitrogen source. The response surface generated based on the second-order coefficient shows that skim milk and yeast extract presented a quadratic effect (Figure 2(c)). The use of skim milk at high concentration with increasing yeast extract concentration (ranging from 1% to 2.8% (wt/vol)) significantly influenced the residual concentration of free amino nitrogen in the culture. On the other hand, reduced residual concentration of free amino nitrogen in the culture was observed at low skim milk concentration (4.00% (wt/vol)) with decreasing yeast extract concentration (ranging from 2.8% to 1% (wt/vol)). This result indicated that interactions between skim milk and yeast extract might have a stronger influence on residual concentration of free amino nitrogen remaining in the culture. Consequently, the use of low skim milk and yeast extract concentration significantly improved the nitrogen uptake efficiency with low residual concentration remaining in the culture at the end of cultivation.

### 3.3. Validation of Optimized Medium

The optimum concentrations for skim milk and yeast extract were 5.89% (wt/vol) and 2.31% (wt/vol), respectively. The responses criteria for the optimization process were maximum for biomass and *β*-galactosidase production. On the other hand, the minimum values were set for residual concentration of lactose and free amino nitrogen. An experiment was performed under the predicted optimal conditions in order to validate the optimized medium. The experimental values fitted well to the predicted results with no significant difference (*P* > 0.05). Therefore, this result was encountered in the process of validation of response surface methodology optimization.

## 4. Conclusion

Results from this study have demonstrated that the optimization of milk-based medium using response surface methodology (RSM) greatly improved G4 cultivation performance, in terms of final cell concentration and *β*-galactosidase as well as residual concentration of lactose and amino nitrogen remaining in the culture. The quadratic model (biomass, *β*-galactosidase production, and free amino nitrogen residual) and linear model (lactose residual) were found sufficient for the optimization of medium for G4. The optimal medium that consists of 5.89% (wt/vol) skim milk and 2.31% (wt/vol) yeast extract gave the final biomass count of 10^7^ cfu/mL, *β*-galactosidase activity of 8.74 U/mL, residual lactose concentration of 9.15 g/L, and residual free amino nitrogen concentration of 226.07 mg/mL. In addition, the use of this optimal medium gave comparable biomass count and *β*-galactosidase activity as those obtained in cultivation using high skim milk concentrations (10% to 13% (wt/vol)). However, residual lactose and free amino nitrogen concentration remaining in the culture at the end of cultivation were reduced by about two times lower as compared to those observed in cultivation using high skim milk concentration.

## Figures and Tables

**Table 1:** The 2^3^ full factorial design and responses for screening experiments.

Run	Factors			Response
	Skim milk (% wt/vol)	Yeast extract (% wt/vol)	Glucose (% wt/vol)	Maximum biomass (log_10_ CFU per milliliter)
1	2 (−1)	0.5 (−1)	0.5 (−1)	4.495
2	2 (−1)	0.5 (−1)	1.5 (+1)	4.789
3	2 (−1)	3 (+1)	0.5 (−1)	5.335
4	2 (−1)	3 (+1)	1.5 (+1)	5.800
5	4 (0)	1.75 (0)	1 (0)	6.417
6	4 (0)	1.75 (0)	1 (0)	6.671
7	4 (0)	1.75 (0)	1 (0)	6.075
8	4 (0)	1.75 (0)	1 (0)	6.824
9	4 (0)	1.75 (0)	1 (0)	6.874
10	6 (+1)	0.5 (−1)	0.5 (−1)	7.158
11	6 (+1)	0.5 (−1)	1.5 (+1)	7.173
12	6 (+1)	3 (+1)	0.5 (−1)	7.204
13	6 (+1)	3 (+1)	1.5 (+1)	7.638

**Table 2:** ANOVA results of the first-order model for 2^3^ full factorial design.

Source of variation or factors	DF^a^	Sum of squares	Mean square	*F* or *t* value	*P* value
Model^b^	7	10.75	1.54	14.09	0.0112
Curvature	7	0.43	0.43	0.071	0.1182
Pure error	4	0.44	0.11	3.94	
Correlation total	12	11.61			

^a^DF: degree of freedom.

^b^ *R* ^2^: 96.10%.

Significant at *α* = 0.05.

**Table 3:** Regression analysis of the 2^3^ full factorial design with maximum biomass (log_10_ cfu/mL) as the response.

Variable	*F* Value	*P* value
*x*_1_^a^	87.94	0.0007
*x*_2_	6.40	0.0647
*x*_3_	1.67	0.2658
*x*_1_ *x*_2_	2.06	0.2246
*x*_1_ *x*_3_	0.11	0.7572
*x*_2_ *x*_3_	0.40	0.5623
*x*_1_ *x*_2_ *x*_3_	0.07	0.8034
Model	14.09	0.0112
Curvature	3.94	0.1182
C.V = 2.78%	*R* ^2^ = 0.96	Adjusted *R* ^2^ = 0.90

^a^ *x*_1_ represents skim milk concentration in % (wt/vol).

*x*_2_ represents yeast extract concentration % (wt/vol).

*x*_3_ represents glucose concentration % (wt/vol).

**Table 4:** The steepest ascent coordination path for all chosen factors at coded and natural levels.

Step	Coded factor^a^		Natural factor^b^ (% wt/vol)		Maximum biomass (log_10_ cfu/mL)^e^
	*ε*_1_	*ε*_2_	*x*_1_	*x*_2_	
Base^c^	0	0	4	1.75	5.77 ± 0.24
Δ^d^	1	0.28	2	0.14	4.05 ± 0.09
Base + Δ	1	0.28	6	1.89	7.68 ± 0.17
Base + 2Δ	2	0.56	8	2.03	7.66 ± 0.68
Base + 3Δ	3	0.84	10	2.17	7.65 ± 0.26
Base + 4Δ	4	1.12	12	2.31	7.63 ± 0.66
Base + 5Δ	5	1.40	14	2.45	7.61 ± 0.04

^a^Coded factor ε_1_: skim milk, ε_2_: yeast extract.

^b^Natural factor x_1_: skim milk, x_2_: yeast extract.

^c^Based: 0 for coded factor and middle point for natural factor.

^d^Movement units (coded factor ε_1_: 0.1/0.1, ε_2_: 0.3/1.09) (natural factor: coded factor x lower point).

^e^Maximum biomass of *B. pseudocatenulatum* G4 (log_10_ cfu/mL) at 21 h of cultivation period. Values are mean of triplicates.

**Table 5:** Experimental design and results using face-centered full factorial design (FCCD).

Run	Skim milk, *x*_1_		Yeast extract, *x*_2_		Responses^a^			
	Coded value	Actual value	Coded value	Actual value	Max. biomass, *y*_1_ (log_10_ cfu/mL)	*β*-Galactosidase production *y*_2_ (U/mL)	Lactose balance *y*_3_ (g/L)	FAN balance *y*_4_ (mg/L)
1	−1	4.0	−1	1.0	5.15	0.19	4.59	198.24
2	−1	4.0	0	1.9	5.18	3.03	4.51	209.58
3	−1	4.0	+1	2.8	5.22	3.13	4.40	213.24
4	0	6.0	−1	1.0	7.09	7.06	10.83	229.15
5	0	6.0	0	1.9	7.15	8.37	11.53	237.06
6	0	6.0	0	1.9	7.11	9.40	11.58	231.04
7	0	6.0	0	1.9	7.12	9.32	11.81	234.65
8	0	6.0	0	1.9	7.14	8.35	11.91	238.98
9	0	6.0	0	1.9	7.16	9.38	11.84	233.14
10	0	6.0	+1	2.8	7.13	8.41	12.90	359.76
11	+1	8.0	−1	1.0	7.26	7.02	22.41	287.53
12	+1	8.0	0	1.9	7.23	6.27	19.10	290.59
13	+1	8.0	+1	2.8	7.11	6.18	25.25	318.70

^a^All factorial and axial points are means of duplicate.

**Table 6:** ANOVA and regression analysis for the response of biomass production (*y*_1_): optimization of skim milk (% wt/vol) and yeast extract (% wt/vol).

Source of variation	Sum of squire or coefficient estimate	DF^a^	MS or Std error	*F* value	*P* value
Model^b^	8.95	4	2.24	2893.35	<0.001
Residual	6.186*E* − 003	8	7.732*E* − 004		
Lack of fit	4.466*E* − 003	4	1.116*E* − 003	2.60	0.1890
Pure error	1.720*E* − 003	4	4.300*E* − 004		
Total	8.95	12			
Factor^c^					
Intercept	7.14	1			
*x*_1_	1.01	1	0.011	7889.68	<0.001^d^
*x*_2_	−6.667*E* − 003	1	0.011	0.34	0.5732
*x*_1_^2^	−0.94	1	0.015	3667.73	<0.001^d^
*x*_1_ *x*_2_	−0.055	1	0.014	15.65	0.0042^d^

^a^DF: degree of freedom.

^b^ *R* ^2^ = 99.93%.

^c^ *x*_1_, skim milk (% wt/vol), *x*_2_, yeast extract (% wt/vol).

^d^Significant at *α* = 0.05.

**Table 7:** Comparison experiments using optimized skim milk and yeast extract based on biomass production, *β*-galactosidase production, lactose, and free amino nitrogen residual at 21 h of cultivation period.

Skim milk (% wt/vol)*	Biomass production (log_10_ cfu/mL)	*β*-Galactosidase production (U/mL)	Lactose (g/L)	Free amino nitrogen (mg/L)
0	1.03 ± 0.11^a∗∗^	0.35 ± 0.21^a^	0.00 ± 0.00^a^	141.57 ± 18.90^a^
4	7.19 ± 0.07^b^	6.36 ± 0.16^b^	7.25 ± 2.02^b^	153.80 ± 15.46^a^
7.17 (optimized concentrations)	7.35 ± 0.06^b^	6.34 ± 0.08^b^	15.92 ± 3.23^c^	198.73 ± 13.67^a^
10	7.78 ± 0.55^b^	5.54 ± 1.02^b^	21.39 ± 3.29^c^	251.57 ± 18.71^b^
12	7.52 ± 0.63^b^	5.23 ± 0.56^b^	25.98 ± 3.19^c^	282.27 ± 11.13^b^
TPY	7.79 ± 0.45^b^	0.05 ± 0.11^a^	0.00 ± 0.00^a^	374.20 ± 16.22^c^

*Cultivation was conducted in different concentrations of skim milk with 1.02% yeast extract supplementation.

**Values in the same column with different letters were significantly different (*P* < 0.05).

**Table 8:** ANOVA analysis for responses *y*_2_ (*β*-galactosidase: U/mL), *y*_3_ (lactose: g/L), and *y*_4_ (FAN: mg/L).

Source	Sum squares	DF	Mean square	*F* value	*P* value
*y*_2_					
Model	96.50	5	19.30	52.91	<0.0001
Residual	2.55	7	0.36		
Lack of fit	1.33	3	0.44	1.46	0.3521
Pure error	1.22	4	0.30		
*R* ^2^ = 0.9742					
*y*_3_					
Model	151.33	2	75.66	615.28	<0.0001
Residual	1.23	10	0.12		
Lack of fit	1.05	6	0.17	3.83	0.1075
Pure error	0.18	4	0.046		
*R* ^2^ = 0.9919					
*y*_4_					
Model	14609.65	5	2921.93	123.58	0.0001
Residual	165.51	7	23.64		
Lack of fit	126.17	3	42.06	4.28	0.0971
Pure error	39.34	4	9.84		
*R* ^2^ = 0.9888					
